# Supplementary material for: DMRforPairs: identifying Differentially Methylated Regions between unique samples using array based methylation profiles
Source: BMC Bioinformatics. 2014 May 15;15:141. doi: 10.1186/1471-2105-15-141 (PMC4046028; doi:10.1186/1471-2105-15-141)
Supplement: Additional file 2 — DMRforPairs output for the comparison of A431-MCF7 and NA17018-NA17105. Please start from the HTML files in each folder. Available via the BMC Bioinformatics website. [file 1471-2105-15-141-S2.zip › 1394847754114233_MOESM2_ESM/A431_MCF7/figures/9751.pdf]

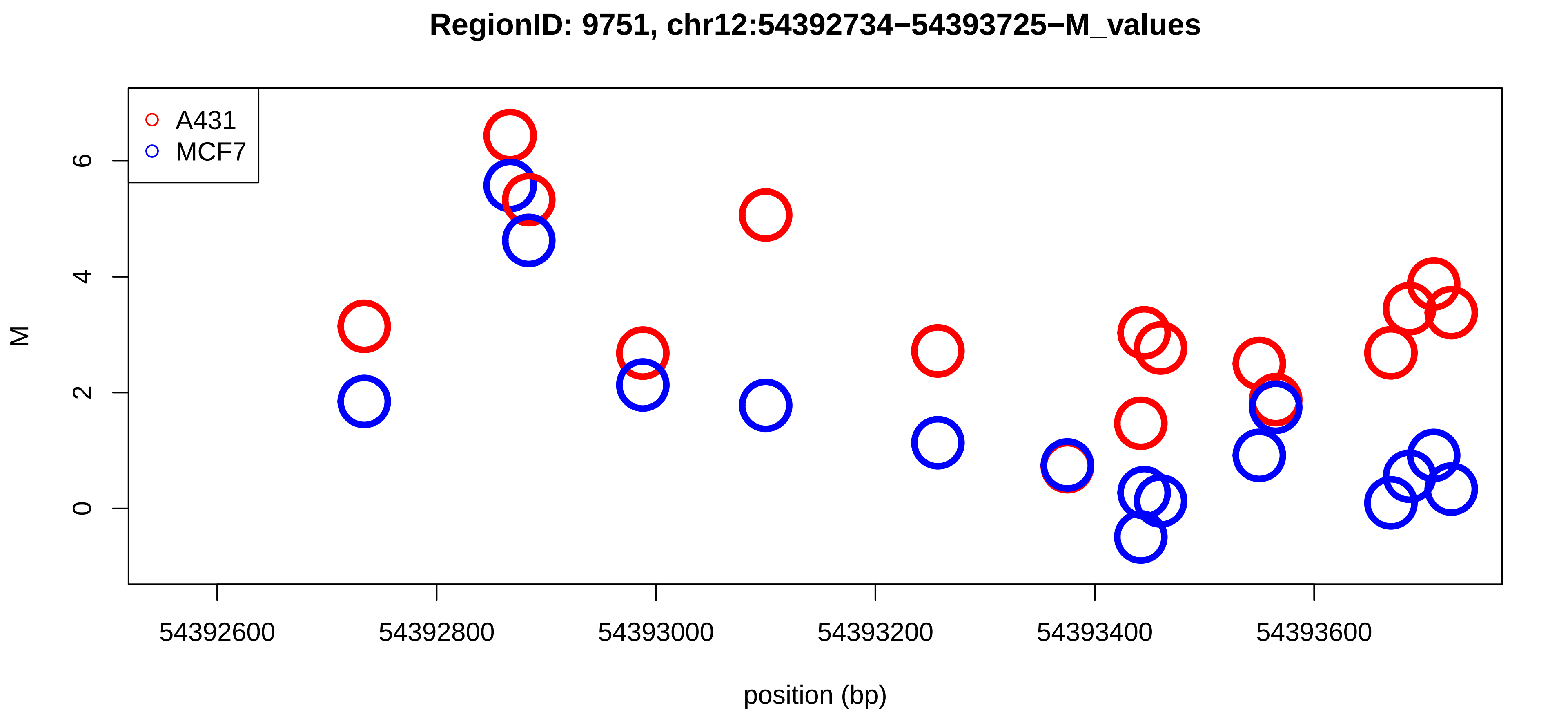
RegionID: 9751, chr12:54392734−54393725−M_values
A431
MCF7
M
0
2
4
6
54392600
54392800
54393000
54393200
54393400
54393600
position (bp)

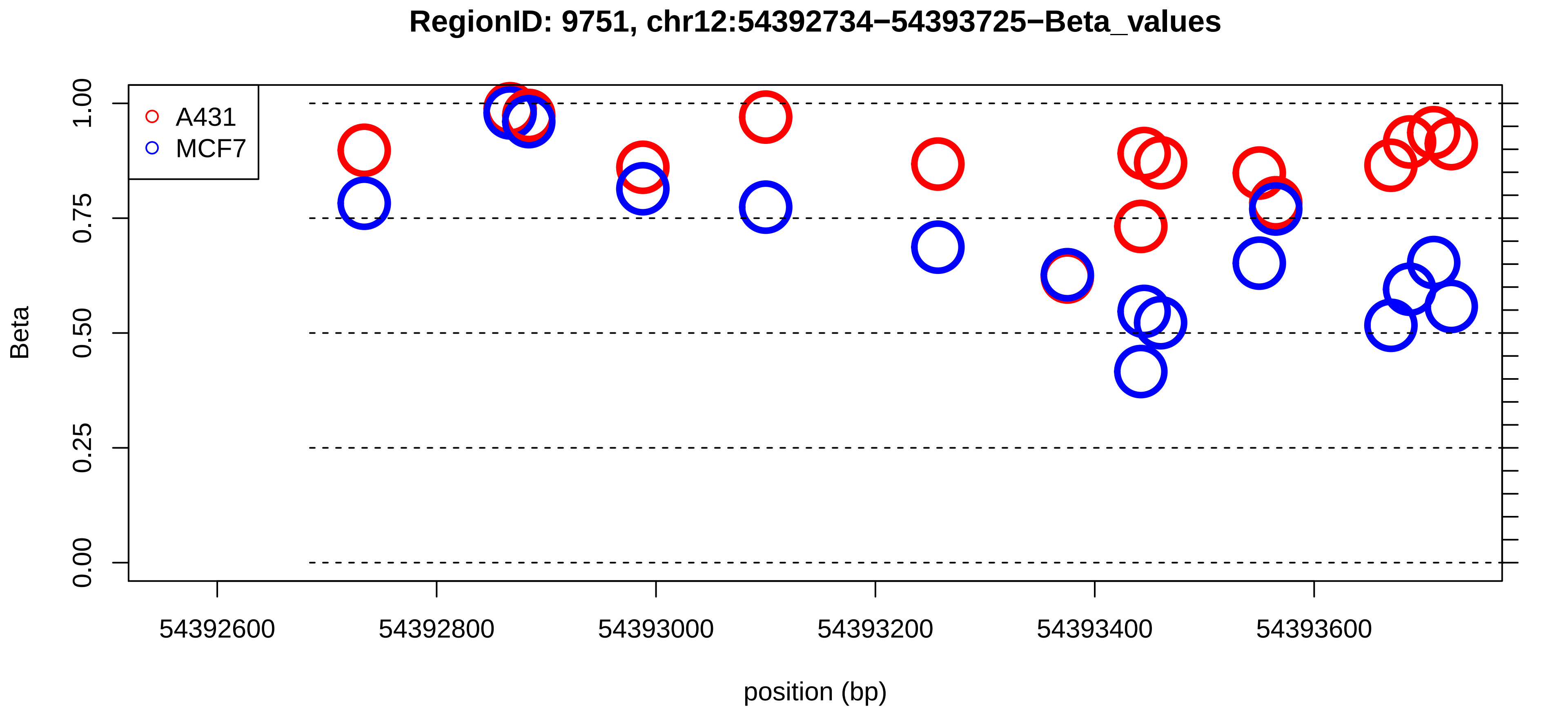
RegionID: 9751, chr12:54392734−54393725−Beta_values
A431
MCF7
Beta
0.00
0.25
0.50
0.75
1.00
54392600
54392800
54393000
54393200
54393400
54393600
position (bp)

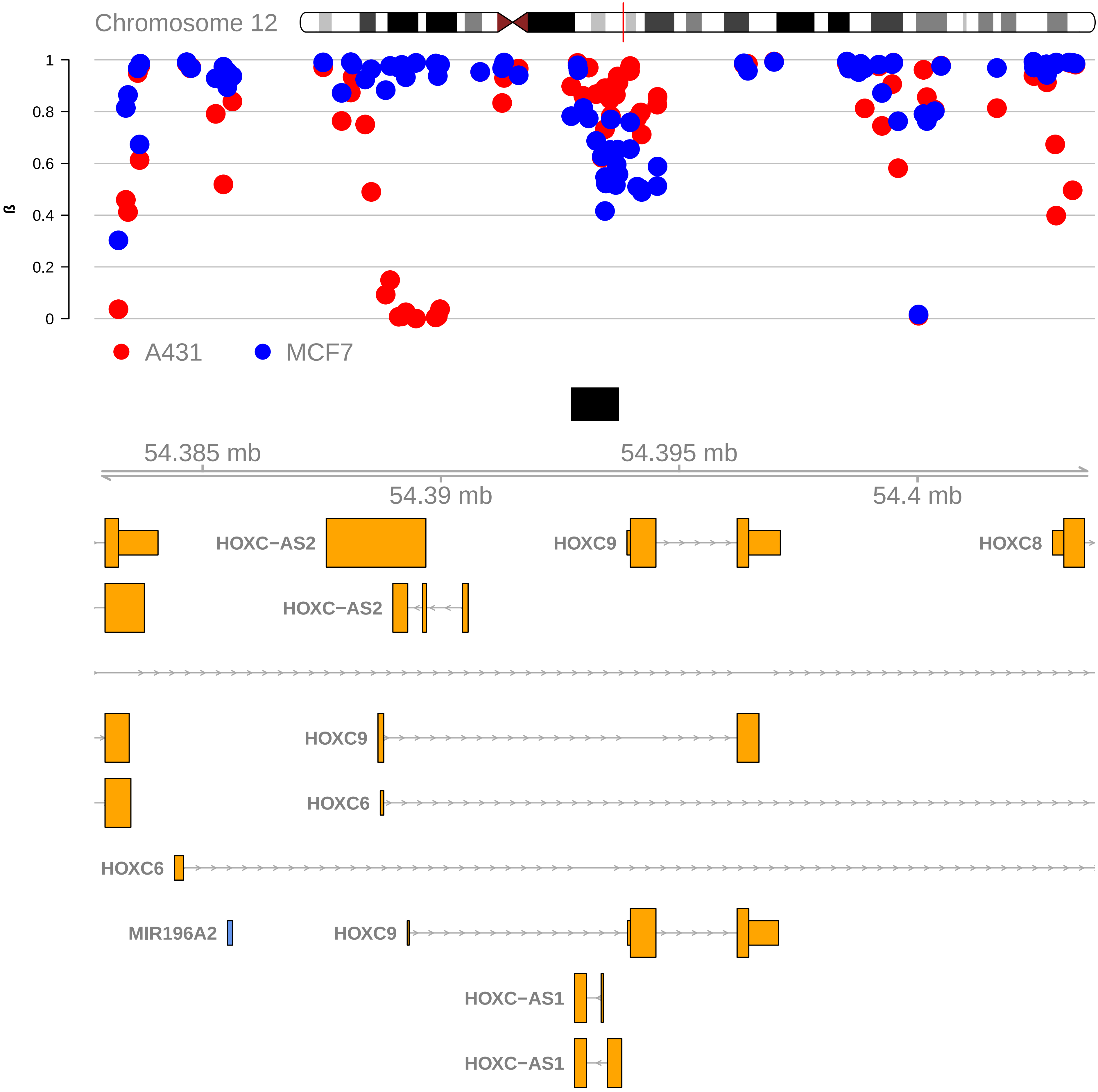
Chromosome 12
1
0.8
0.6
0.4
0.2
0
β
A431
MCF7
54.385 mb
54.395 mb
54.39 mb
54.4 mb
HOXC−AS2
HOXC9
HOXC8
HOXC−AS2
HOXC9
HOXC6
HOXC6
MIR196A2
HOXC9
HOXC−AS1
HOXC−AS1
